# Optimization of Multiple Pathogen Detection Using the TaqMan Array Card: Application for a Population-Based Study of Neonatal Infection

**DOI:** 10.1371/journal.pone.0066183

**Published:** 2013-06-21

**Authors:** Maureen H. Diaz, Jessica L. Waller, Rebecca A. Napoliello, Md. Shahidul Islam, Bernard J. Wolff, Daniel J. Burken, Rhiannon L. Holden, Velusamy Srinivasan, Melissa Arvay, Lesley McGee, M. Steven Oberste, Cynthia G. Whitney, Stephanie J. Schrag, Jonas M. Winchell, Samir K. Saha

**Affiliations:** 1 Respiratory Diseases Branch, Division of Bacterial Diseases, Centers for Disease Control and Prevention, Atlanta, Georgia, United States of America; 2 Department of Microbiology, Bangladesh Institute of Child Health, Child Health Research Foundation, Dhaka Shishu Hospital, Dhaka, Bangladesh; 3 Division of Preparedness and Emerging Infections, Centers for Disease Control and Prevention, Atlanta, Georgia, United States of America; 4 Polio and Picornavirus Laboratory Branch, Division of Viral Diseases, Centers for Disease Control and Prevention, Atlanta, Georgia, United States of America; Naval Research Laboratory, United States of America

## Abstract

Identification of etiology remains a significant challenge in the diagnosis of infectious diseases, particularly in resource-poor settings. Viral, bacterial, and fungal pathogens, as well as parasites, play a role for many syndromes, and optimizing a single diagnostic system to detect a range of pathogens is challenging. The TaqMan Array Card (TAC) is a multiple-pathogen detection method that has previously been identified as a valuable technique for determining etiology of infections and holds promise for expanded use in clinical microbiology laboratories and surveillance studies. We selected TAC for use in the Aetiology of Neonatal Infection in South Asia (ANISA) study for identifying etiologies of severe disease in neonates in Bangladesh, India, and Pakistan. Here we report optimization of TAC to improve pathogen detection and overcome technical challenges associated with use of this technology in a large-scale surveillance study. Specifically, we increased the number of assay replicates, implemented a more robust RT-qPCR enzyme formulation, and adopted a more efficient method for extraction of total nucleic acid from blood specimens. We also report the development and analytical validation of ten new assays for use in the ANISA study. Based on these data, we revised the study-specific TACs for detection of 22 pathogens in NP/OP swabs and 12 pathogens in blood specimens as well as two control reactions (internal positive control and human nucleic acid control) for each specimen type. The cumulative improvements realized through these optimization studies will benefit ANISA and perhaps other studies utilizing multiple-pathogen detection approaches. These lessons may also contribute to the expansion of TAC technology to the clinical setting.

## Introduction

Identifying infectious etiologies is extremely complex for many clinical syndromes. The diversity and fastidious nature of pathogens causing infection and the lack of available diagnostic tests of sufficient quality and breadth contribute to the low proportion of cases with clearly identified etiology. The infectious etiology of community-acquired respiratory infections may remain unidentified in a large proportion of cases, even when advanced diagnostic technologies are employed [1]–[5]. In resource-poor settings, this proportion may be even higher as a result of factors which impede utilization of sophisticated testing procedures, including logistical challenges to specimen collection and handling, as well as the lack of adequate laboratory space or highly trained personnel. Obtaining an appropriate high-quality specimen at the proper time can be especially challenging within certain populations due to limited care-seeking or inadequate access to quality materials. High potential for contamination in non-sterile collection settings is another considerable challenge to obtaining quality results in resource-poor areas. Interpretation of results from respiratory specimens is an additional challenge since simply confirming the presence of a microorganism does not indicate whether it is etiologic. Many potential pathogens are carried asymptomatically in a proportion of individuals within a population. Furthermore, when more than one potential pathogen is identified, determining which organism(s) contributes to disease can be difficult.

Nucleic acid detection methods have increasingly been replacing or augmenting traditional laboratory techniques for identification of etiology of infectious disease syndromes. Multiple pathogen detection approaches which incorporate these highly sensitive methods into high-throughput strategies are being implemented as tools for identification of etiology in clinical syndromes where many infectious agents may contribute to disease [6]. The increased availability of these multi-pathogen molecular detection methods enables a more comprehensive evaluation of patient specimens in a relatively short period of time, resulting in an improved clinical picture for consideration by the physician. One technology which has emerged as a useful method for multiple pathogen detection is the TaqMan Array Card (TAC), formerly known as the TaqMan Low Density Array (TLDA) card (Life Technologies, Foster City, CA, USA). This 384-well microfluidic array consists of dried-down individual singleplex qPCR reactions for simultaneous detection of up to 48 targets from a single specimen. Each TAC contains eight individual microfluidic channels that can be loaded with PCR reactions containing nucleic acid extract from a clinical specimen or control material. TAC technology has previously been evaluated for detection of respiratory pathogens [7], enteropathogens [8], and biothreat agents [9], [10], and has been routinely employed to determine etiology of respiratory disease outbreaks in the United States [11].

Recently, this approach has been proposed as an effective strategy for identifying disease etiologies in large-scale surveillance studies [8], [12]. TAC was selected for use in the Aetiology of Neonatal Infection in South Asia (ANISA) study, supported by the Bill and Melinda Gates Foundation (BMGF). This study aims to address the critical deficiency in childhood survival in South Asia, a region of the world where pneumonia, sepsis, and diarrheal disease cause the majority of childhood deaths, over 40% of which occur in neonates [13]. The current rate of decline in mortality in this region is not sufficient to meet the target of the United Nations Millennium Development Goal 4 (MDG 4) of a two-thirds reduction in child mortality by 2015 [14]. The goals of the ANISA study are to determine the population-based incidence, etiology, and characteristics of community-acquired infections and identify associated risk factors in neonates in Bangladesh, India, and Pakistan. As part of this study, respiratory and blood specimens will be tested using both traditional and advanced diagnostic techniques in order to identify the leading causes of neonatal infections in these countries. TAC was selected for use in this project based upon (i) prior application of this technology for pathogen detection, (ii) ability to customize a panel of pathogen targets, (iii) requirement for minimal volume of specimen, (iv) reduced potential for contamination due to the closed system format of TAC, and (v) ease of use, including minimal “hands-on” setup.

Despite its favorable advantages, a number of technical challenges existed for application of TAC to the ANISA study. First, the utility of TAC for pathogen detection in human blood specimens has not previously been investigated. A recent study demonstrated low sensitivity of TAC for detection of several bacterial agents in blood from murine infection models [10]. Molecular detection of pathogens in blood suffers from poor sensitivity, and blood volumes collected from neonates are limited. Therefore, inclusion of this specimen type in the ANISA study required modification and optimization of the nucleic acid extraction protocol as well as other experimental parameters in order to improve the sensitivity of this technology for pathogen detection in blood. Secondly, the diversity of pathogens, including viruses, bacteria, and parasites, created an additional challenge since many of these pathogen-specific assays had not been designed and/or evaluated for performance on the TAC. The diverse repertoire of pathogens included both DNA and RNA genomes, requiring a reverse transcription step for all assays due to the integrated format of TAC. Therefore, assays designed to detect DNA targets also required optimization with RT-qPCR cycling conditions and enzyme formulations.

Here we report data from a series of experiments designed to optimize the TAC methodology for use in the ANISA study. Improvements include enhancement of nucleic acid extraction from blood using an initial lysis step, implementation of an improved RT-qPCR enzyme system, and optimization of card design and production. The results from these optimization studies are being applied to TAC testing procedures in the ANISA study in order to achieve the highest sensitivity and specificity possible for determining infectious etiologies among enrolled neonates. In some cases, the findings reported here may also be applicable to studies using other specimen types along with testing modalities that may hold utility within the clinical setting.

## Materials and Methods

### Ethics Statement

The study protocols were approved by the International Centre for Diarrhoeal Disease Research, Bangladesh (IDCCR,B, Dhaka, Bangladesh) and Aga Khan University (Karachi, Pakistan). Written informed consent was obtained from a parent or guardian prior to specimen collection from neonate.

### Real-time PCR Assay Design and Analytical Validation

**Figure 1:**
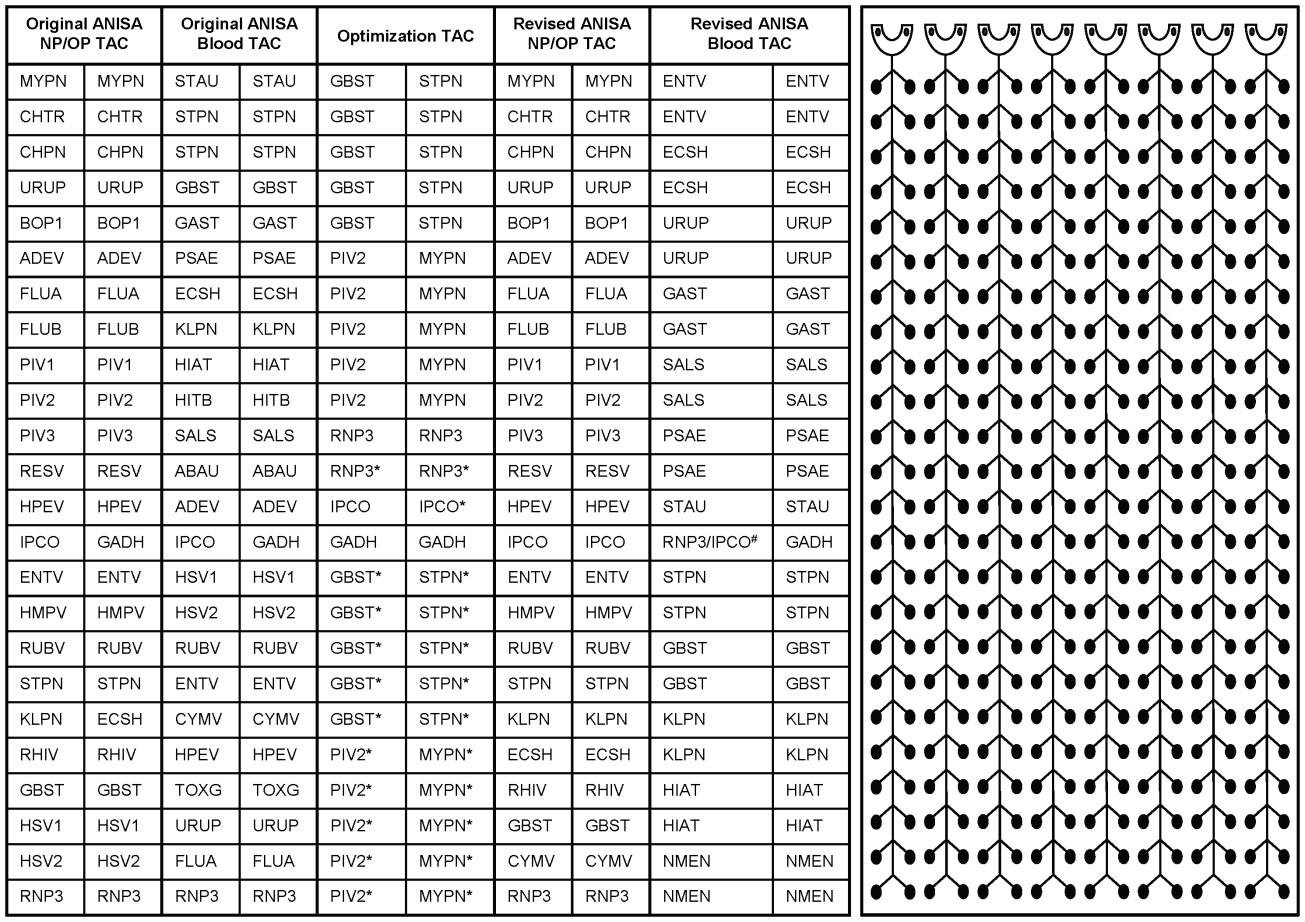
ANISA-specific TAC configurations. Original ANISA NP/OP TAC, Original ANISA Blood TAC, Optimization TAC, Revised ANISA NP/OP TAC, Revised ANISA Blood TAC. Target designations include: MYPN, *Mycoplasma pneumoniae*; CHTR, *Chlamydia trachomatis*; CHPN, *Chlamydophila pneumoniae*; URUP, *Ureaplasma* spp.; BOP1, *Bordetella pertussis*; ADEV, Adenovirus; FLUA, Influenza A; FLUB, Influenza B; PIV1, Parainfluenza virus 1; PIV2, Parainfluenza virus 2; PIV3, Parainfluenza virus 3; RESV, Respiratory Syncytial Virus; HPEV, Human Parechovirus; IPCO, Internal Positive Control; GADH, Glyceraldehyde phosphate dehydrogenase (Manufacturing control); ENTV, Enterovirus; HMPV, Human Metapneumovirus; RUBV, Rubella virus; STPN, *Streptococcus pneumoniae*; KLPN, *Klebsiella pneumoniae*; ECSH, *Escherichia coli*/*Shigella* spp.; RHIV, Rhinovirus; GBST, Group B *Streptococcus*; HSV1, Herpes Simplex Virus 1; HSV2, Herpes Simplex Virus 2; RNP3, Human RNaseP; STAU, *Staphylococcus aureus*; GAST, Group A *Streptococcus*; PSAE, *Pseudomonas aeruginosa*; HIAT, *Haemophilus influenzae*; HITB, *Haemophilus influenzae* type B; SALS, *Salmonella* spp.; ABAU, *Acinetobacter baumannii*; CYMV, Cytomegalovirus; TOXG, *Toxoplasma gondii*; NMEN, *Neisseria meningitidis*. All oligonucleotides were spotted at 1× final concentration except where noted by * where concentration is 2×. ^#^Duplex assay consisting of RNP3 assay with FAM-labeled probe and IPCO assay with VIC-labeled probe.

A panel of neonatologists with expertise in neonatal infection and South Asia was convened, and the Delphi method [15] was used to identify organisms of the highest priority for testing in nasopharyngeal (NP) and oropharyngeal (OP) swabs and blood specimens from neonates enrolled in the ANISA study. Primers and hydrolysis probes were designed for specific detection of organisms for which existing assays were not currently available in our laboratories, including *Toxoplasma gondii*, *Staphylococcus aureus*, *Klebsiella pneumoniae*, *Escherichia coli*/*Shigella* spp., *Pseudomonas aeruginosa*, *Ureaplasma* spp., *Chlamydia trachomatis*, *Acinetobacter baumannii*, *Streptococcus agalactiae* (Group B *Streptococcus*), and *Neisseria meningitidis* (Table S1 in File S1). In most cases, oligonucleotides were designed using Primer Express 3.0 software (Applied Biosystems, Foster City, CA) with slight modifications to optimize melting temperatures (Tm) and minimize intra- and inter-molecular interactions. Specificity of each set of oligonucleotides for the intended genus and/or species was assessed by sequence comparison using Basic Local Alignment Search Tool (BLAST) within the National Center for Biotechnology Information (NCBI) database (http://blast.ncbi.nlm.nih.gov/Blast.cgi). Primers and hydrolysis probe previously reported for detection of *Salmonella* spp. were validated using the experimental conditions for this study (Table S1 in File S1) [16]. Performance evaluation of other assays included on the ANISA study TAC designs (Figure 1) was reported previously [7]. Primers and hydrolysis probes used for analytical validation were manufactured by the Biotechnology Core Facility at the Centers for Disease Control and Prevention (CDC, Atlanta, GA, USA).

All newly developed real-time PCR assays were evaluated using individual RT-qPCR reactions prior to use on the TAC format. Each assay was tested using nuclease-free water as template (*n* ≥95) to ensure no fluorescence amplification signal was observed in the absence of nucleic acid. The limit of detection was independently determined for each assay by testing at least 3 replicates each of a 10-fold dilution series of specific total nucleic acid ranging from 0.1 fg/µL to 1 ng/µL. Inclusivity was assessed by testing representative isolates, including various subspecies, serotypes, or clonal groups, as appropriate (Table S2 in File S1). Specificity of each assay was assessed by testing 15 ng of nucleic acid from at least 200 different bacteria, viruses, and protozoa representing 36 genera and 143 species. In addition to the most closely related species to each target pathogen, this panel also included commensals of the respiratory tract and human nucleic acid.

### Oligonucleotide Preparation for TAC Manufacturing

Oligonucleotides for TAC production were manufactured by Integrated DNA Technologies (Coralville, IA, USA) or Biosearch Technologies (Novato, CA, USA), diluted and combined to 20× reaction concentration, and provided to Life Technologies (Foster City, CA, USA) for custom manufacturing of study-specific TACs (Figure 1). The 20× concentration corresponds to a 1× final reaction concentration in the 1 µL reaction within each TAC well on the finished array card. For optimization of primer and probe concentration, oligonucleotides were also prepared at 40× concentration for TAC production, resulting in 2× final reaction concentration in each well. The Optimization TAC configuration (Figure 1) was designed to assess the impact of oligonucleotide concentration on pathogen detection and consisted of five replicates of four targets (*M. pneumoniae*, *S. pneumoniae,* Group B *Streptococcus*, and human parainfluenza virus 2), each spotted in two final reaction concentrations, 1× and 2× (Figure 1). Total nucleic acid was extracted from a series of 10-fold dilutions of each organism and tested to determine the potential impact of oligonucleotide concentration on assay sensitivity.

### Clinical Specimens

Clinical specimens, including whole blood and combined NP/OP swabs, were obtained from enrolled neonates at three ANISA study sites: Sylhet, Bangladesh; Karachi, Pakistan; and Matiari, Pakistan. NP/OP swabs were collected and placed together in 1 mL Universal Transport Media (UTM, Copan Diagnostics, Murrieta, CA, USA) and stored at −70°C prior to extraction. Blood specimens were collected in standard EDTA collection tubes and stored at 4°C for short-term storage (≤72 h post-collection) or −70°C for longer storage prior to nucleic acid extraction and testing by TAC.

### Specimen Processing and Nucleic Acid Extraction

Total nucleic acid (TNA) was extracted from clinical specimens using the MagNA Pure Compact instrument (Roche Applied Sciences, Indianapolis, IN, USA) with Nucleic Acid Isolation Kit I and Total NA Plasma protocol. For NP/OP swab specimens, 400 µL of UTM was extracted and eluted in 100 µL. For extraction of whole blood, 300 µL of blood in EDTA was mixed with 100 µL of a freshly-prepared solution of lytic enzymes consisting of 1.5 mg/mL lysostaphin, 2500 U/mL mutanolysin, and 200 mg/mL lysozyme (Sigma-Aldrich, St. Louis, MO, USA) in Tris-EDTA (TE) buffer and incubated at 37°C for 60 min. prior to extraction on the MagNA Pure Compact, with elution in 100 µL. To assess the potential impact of an initial lysis step on recovery of TNA from a variety of pathogens, healthy donor blood was spiked with 10-fold serial dilutions of quantified culture stock of gram-positive bacteria (*Streptococcus pyogenes* or *S*. *aureus)*, gram-negative bacterium (*K. pneumoniae)*, or an RNA virus (enterovirus) and tested using individual RT-qPCR reactions. Spiked blood specimens were extracted directly or incubated with TE buffer or TE buffer containing lytic enzymes at 37°C for 60 min. prior to extraction. Ct values for spiked blood experiments were compared using Student’s two-tailed *t* test.

### Individual Real-time PCR Assay Performance

All individual real-time PCR assays were performed on the Applied Biosystems 7500 Real-Time PCR system (Life Technologies, Foster City, CA, USA) with the following cycling conditions: 45°C for 10 min, 94°C for 10 min, 45 cycles of 94°C for 30 s and 60°C for 60 s, with data acquisition in the FAM channel during the 60°C step. Each reaction consisted of 1× AgPath-ID One-step RT-PCR buffer and 1× AgPath-ID One-step RT-PCR enzyme mix (Applied Biosystems, Foster City, CA, USA) or 1× qScript XLT One-step RT-qPCR ToughMix, low ROX (Quanta Biosciences, Gaithersburg, MD, USA), forward and reverse primers and FAM-labeled hydrolysis probe at the concentrations listed in Table S1 in File S1, and nuclease-free water to final volume of 20 µL. Five µL of TNA was used in each reaction.

### TAC Assay Performance

TAC assays were performed with slight modification of the previously reported procedure [7]. Briefly, mastermix for each TAC consisted of the following: 1× AgPath-ID One-step RT-PCR buffer and enzyme or 1× qScript XLT One-step RT-qPCR ToughMix. Reactions tested using AgPath enzyme system consisted of 50 µL 2× buffer, 4 µL 25× enzyme mix, and 46 µL of TNA. Reactions tested with the qScript ToughMix enzyme system consisted of 50 µL 2× mastermix and 50 µL of TNA. Each card was centrifuged at 336 × *g* for 1 min. twice, to distribute the fluid in the reaction wells, and sealed to sequester individual reactions. All TACs were run on the Applied Biosystems ViiA7 Real-Time PCR system (Life Technologies, Foster City, CA, USA) using the same cycling conditions as used for individual RT-qPCR reactions. A no template control (NTC) and a positive control consisting of combined RNA transcripts generated as previously described [17] were included on each TAC.

## Results

### Analytical Validation of New Real-time PCR Assays

For each new assay, no amplification was observed in no-template control (NTC) reactions (*n* ≥95) or in reactions containing nucleic acid from other organisms (*n* ≥200, data not shown). Each assay was also tested for inclusivity within the genus or species using representative isolates of each subspecies or serotype as appropriate (Table S2 in File S1). The number of isolates used for inclusivity testing varied based on availability. The limit of detection was independently determined for each assay (Table S2 in File S1).

The target for real-time PCR detection of *E. coli* also reacts with the *Shigellae*. This *E. coli/Shigella* assay successfully detected all *E. coli* types tested, including representative isolates of each virotype (EHEC, EPEC, ETEC, EAEC, and EIEC), but did not amplify the closely related species *E. albertii, E. hermannii,* or *E. fergusonii* (data not shown). This assay also detected all four *Shigella* species (*S. flexneri*, *S. sonnei*, *S. dysenteriae*, and *S. boydii*), with the exception of *S. dysenteriae* serotype I (data not shown). During development and validation of this assay, we occasionally observed sporadic amplification signal in NTC reactions. This was determined to occur due to residual *E. coli* DNA present in the enzyme preparation from the manufacturers, which varied between production lots. Residual *E. coli* DNA in extraction reagents may also contribute to this phenomenon during testing of clinical specimens. Crossing threshold (Ct) values for this sporadic amplification were generally found to be >30. For this reason, we chose to implement a Ct cutoff value of 30 for a more accurate interpretation of this assay.

### Extraction of TNA from Blood and Saline

**Figure 2:**
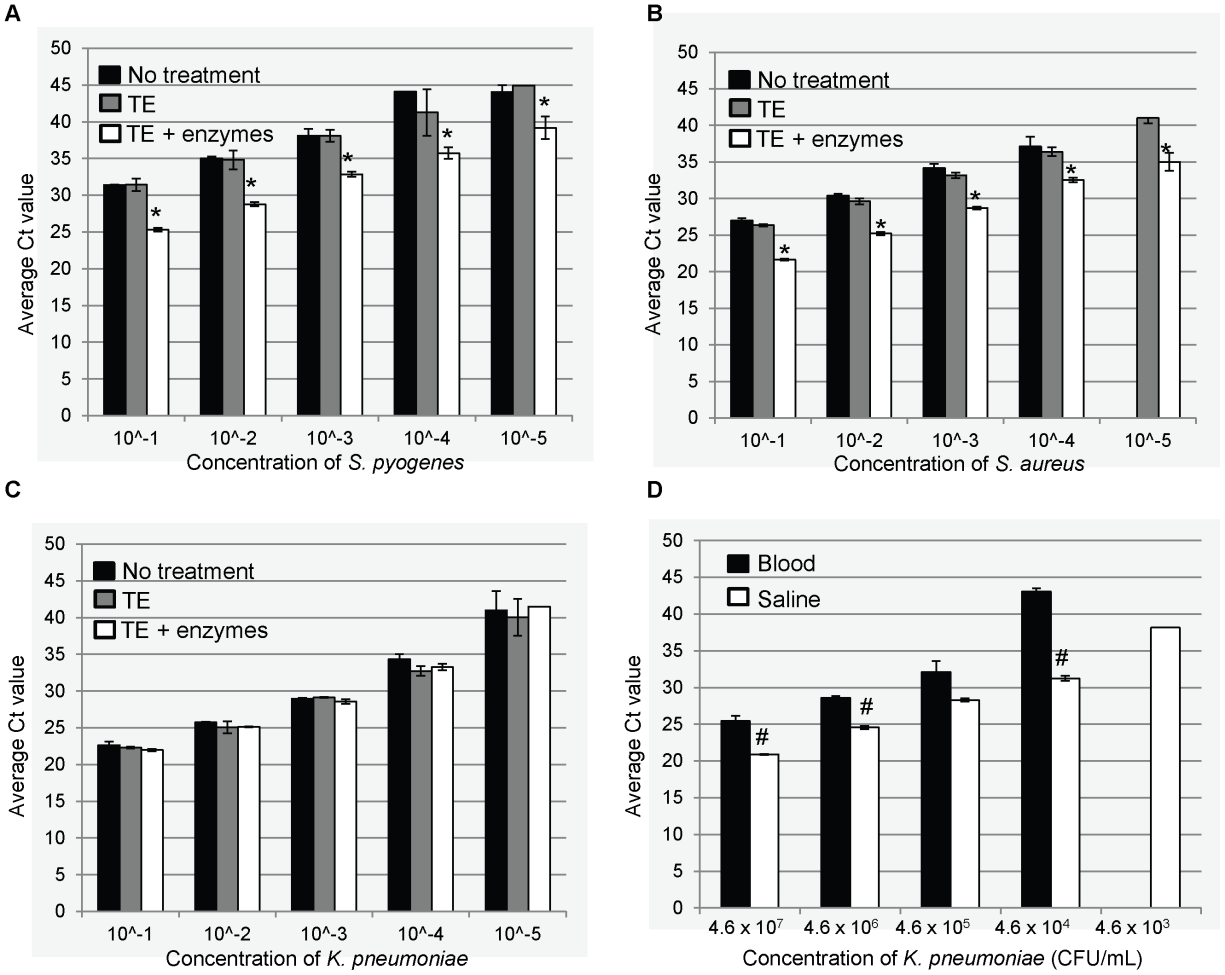
Effect of lytic enzyme treatment on extraction of nucleic acid from blood specimens. Average Ct value of individual real-time PCR reactions (*n* = 4) containing TNA extracted from healthy donor blood spiked with serial dilutions of *S. pyogenes* (A), *S. aureus* (B), or *K. pneumoniae* (C) without treatment or after incubation with TE buffer alone or TE buffer with lytic enzymes (lysozyme, lysostaphin, and mutanolysin) at 37°C for 30 min. (D) Ct values of serial dilutions of *K. pneumoniae* spiked into saline (to mimic NP/OP swab) or blood. Error bars display standard deviation. *p<0.0001 compared to no treatment. ^#^ *p*<0.05 compared to same concentration of organisms in saline.

Direct comparison of Ct values of TNA extracted from blood spiked with gram-positive bacteria (*S. pneumoniae* or *S. aureus*) revealed that the average Ct value was approximately 5.5 cycles lower for TNA extracted after incubation with a lytic enzyme solution compared to identical preparations without this pre-treatment step (Figure 2A–B). Incubation of spiked blood specimens with TE buffer alone did not result in lower Ct values, indicating that the observed improvement in Ct values is a result of the enzyme treatment instead of simply dilution and heating (Figure 2). Pre-treatment with lytic enzymes had no significant impact on Ct values of TNA from blood spiked with the gram-negative bacterium *K. pneumoniae* (Figure 2C) or enterovirus (data not shown).

In addition, the initial lysis step did not significantly impact target detection in saline spiked with the same serial dilutions (data not shown). However, comparison of the Ct values for TNA extracted from blood and saline spiked with the same concentration of organisms revealed that detection in blood is significantly impaired relative to saline. The difference in Ct value for detection of the same number of bacteria in blood compared to saline ranged from 3.8 cycles at higher concentrations to 11.8 cycles at lower concentrations (mean difference in Ct value = 6), including a complete lack of detection of the lowest concentration in blood (Figure 2D). These data demonstrate the inherent challenge of detecting pathogens in whole blood specimens.

### TAC Preparation: Oligonucleotide Concentration and Assay Replicates

The impact of oligonucleotide concentration on pathogen detection was briefly examined using the optimization TAC configuration (Figure 1). No significant difference in target amplification was observed at the limit of detection for any of the four targets examined (data not shown). Additionally, testing of clinical specimens previously known to be positive for each of these pathogens did not reveal any oligonucleotide concentration-dependent difference in target detection (data not shown).

We next aimed to determine the optimal number of replicates per target needed to maximize pathogen detection, particularly when the concentration of nucleic acid was near the limit of detection of the assay. Testing of serial dilutions of nucleic acid from *M. pneumoniae*, *S. pneumoniae*, *S. agalactiae*, and PIV2 revealed excellent concordance between replicates at higher TNA concentrations. In contrast, the number of replicate reactions in which amplification was observed decreased as the concentration approached the limit of detection for each assay. Testing five replicates allowed detection of less concentrated nucleic acid compared to two replicates (data not shown). These results indicate that testing a higher number of replicates improves pathogen detection rates when the concentration of organisms is near the limit of detection.

**Figure 3:**
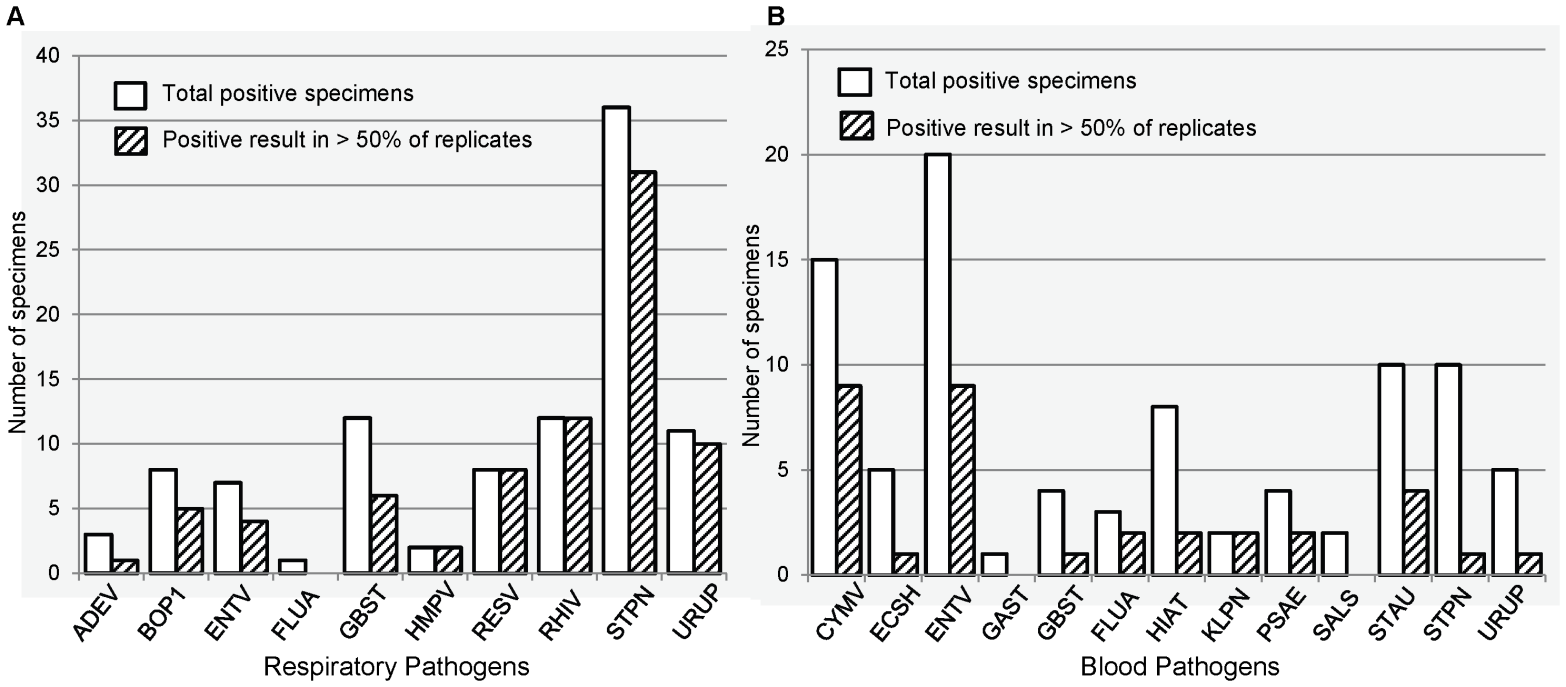
Concordance between replicates of primary clinical specimens tested on TAC. Concordance between replicate results for NP/OP (A) and blood (B) specimens tested using TAC. Data shown are total number of specimens identified as positive in at least one replicate reaction (white bars) and proportion of positive specimens for which greater than 50% of replicates were positive (shaded bars). Number of replicates tested varied by target and specimen type; all targets were tested in ≥2 replicates. Total number of specimens tested, NP/OP (*n* = 124), blood (*n* = 661).

We also tested primary NP/OP swab and blood specimens in order to assess the value of testing additional replicates on pathogen detection. Positive results in clinical specimens were confirmed by repeat testing in individual RT-qPCR reactions followed by confirmation of the appropriate size amplicon (data not shown). The proportion of NP/OP and blood specimens identified as positive in more than half of assay replicates varied by target (Figure 3). Overall, this proportion was significantly higher in NP/OP specimens (Figure 3A) compared to whole blood (Figure 3B). In other words, while the majority of NP/OP specimens that were positive were identified as positive in more than half of assay replicates, amplification of pathogen-specific targets in whole blood extracts occurred in a much smaller proportion of total replicates tested. Furthermore, average Ct values were higher in positive reactions containing TNA extracted from blood compared to NP/OP swabs (data not shown). Together these data suggest that a higher number of assay replicates may identify pathogens which otherwise would be missed, particularly in whole blood specimens.

### Enzyme System Performance with TAC

**Figure 4:**
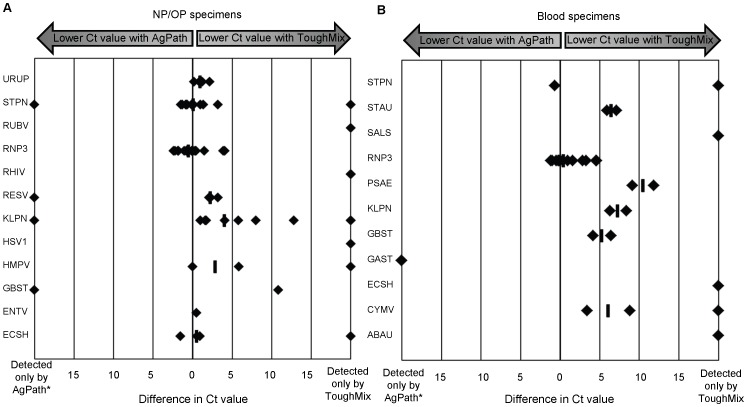
Effect of enzyme system on detection of pathogen targets in primary clinical specimens. Data shown are difference in Ct value between reactions using Quanta One-step RT-PCR ToughMix and AgPath-ID One-step RT-PCR kit when testing TNA extracted from NP/OP swabs (A) or blood (B). Each data point represents the difference in Ct value between the two reactions for an individual clinical specimen. Median difference is indicated (**―**) for assays with ≥2 positive results. *Targets that were only detected using AgPath always occurred when Ct values were >33.

Finally, we assessed whether different enzyme chemistries affected target detection in primary clinical specimens. We hypothesized that newer generations of enzyme mixes may result in improved detection of pathogen targets, particularly in the presence of molecules known to have inhibitory effects on real-time PCR, such as some blood components. We compared the performance of two enzyme formulations, Ambion AgPath-ID One-step RT-PCR kit (Applied Biosystems) and Quanta qScript XLT One-step RT-qPCR ToughMix, low ROX (Quanta Biosciences), at detecting targets in NP/OP specimens (*n* = 18) and blood specimens (*n* = 12) (Figure 4). The median difference in Ct value for positive results in NP/OP specimens tested with ToughMix compared to AgPath enzyme varied by target (range 0–4), but overall improved Ct values were observed in reactions using ToughMix enzyme (Figure 4A). An even more dramatic improvement in Ct values was observed for blood specimens tested with ToughMix compared to AgPath; the median difference in Ct value ranged from 0.46 to 10.5 for various targets (Figure 4B). In addition, we detected additional positive results (*n* = 16) for pathogen-specific targets using the ToughMix enzyme mix that were not detected when testing the same specimen extract using AgPath. This phenomenon was not limited to a single pathogen target, but rather occurred with 12 unique assays (Figure 4). While a few instances (*n* = 5) were also observed where the reactions using AgPath yielded a positive result while the ToughMix reaction was negative, this occurred only when the Ct value with AgPath was >33, at the threshold where reproducibility between replicates is most commonly discordant. Overall, improved pathogen detection was observed using the ToughMix enzyme system, particularly in primary blood specimens.

## Discussion

**Figure 5:**
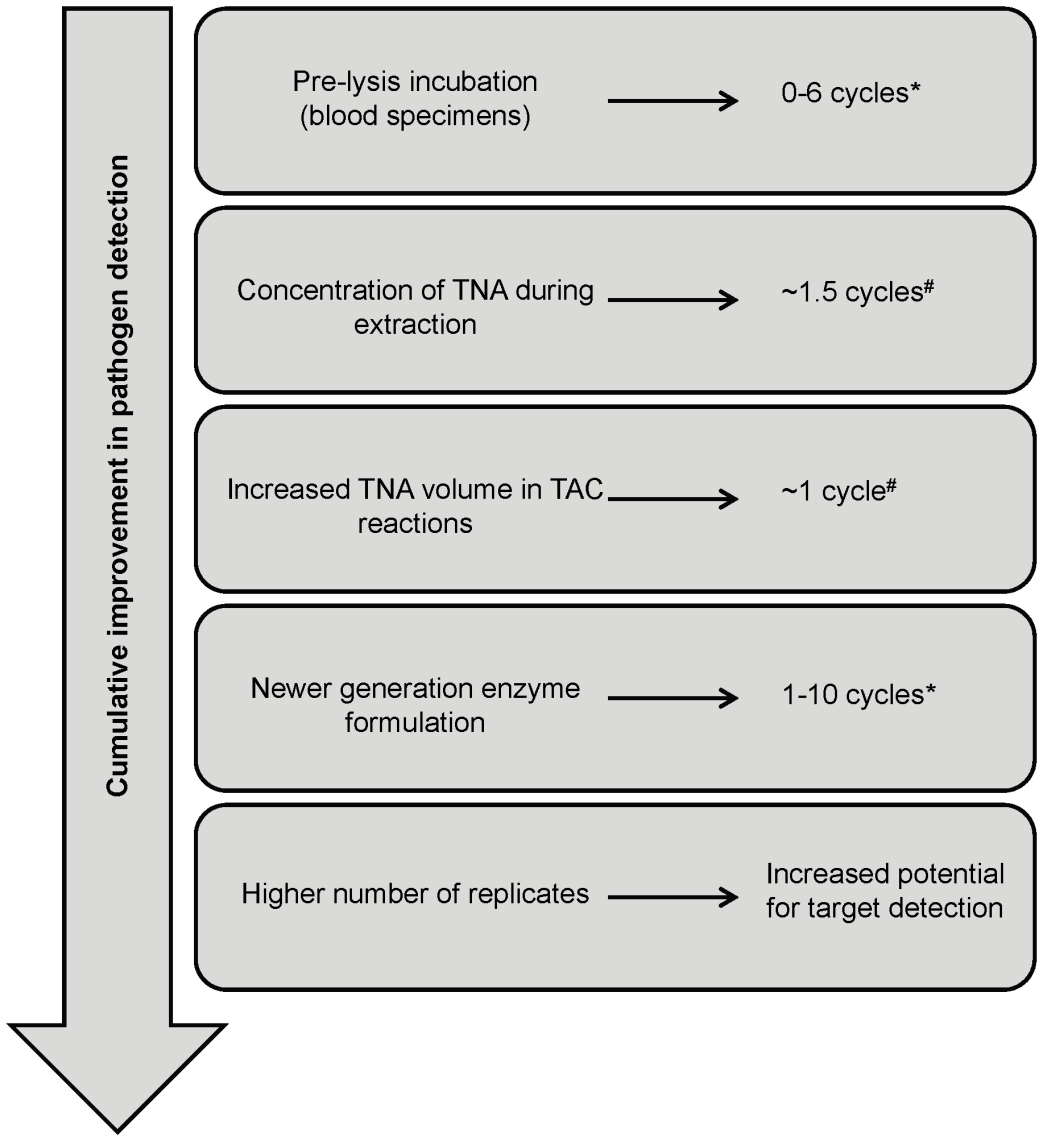
Cumulative improvement of pathogen detection with optimization of experimental parameters. Potential improvement in Ct value (number of cycles) achieved by optimizing each experimental parameter. *Number of cycles gained varies based on organism, target, and specimen type. ^#^Theoretical improvement calculated based on assumption of 3.3 cycle difference with 10-fold change in nucleic acid concentration.

The availability of multiple-pathogen detection technologies has the potential to improve the identification of etiology in a variety of infectious disease syndromes, ultimately resulting in a more comprehensive clinical evaluation. The TAC method has recently been implemented for broad pathogen detection and has proven to be a valuable tool for identification of infectious etiology [7], [8], [11]. Building upon existing data, we sought to improve the performance of TAC through procedural modifications in order to enhance pathogen detection for use in a surveillance study of neonatal infections in South Asia. Experiments were conducted to optimize performance of this technology, including nucleic acid extraction, TAC design and production, and use of a newer generation enzyme mix. We show that considerable improvement can be achieved by targeted optimization of experimental parameters (Figure 5). The experiments performed here specifically address the need for optimization of the entire TAC testing procedure in order to achieve the highest quality data, a need which has been recognized by other investigators utilizing TAC for pathogen detection [10]. These findings may also be applicable when implementing TAC or other multiple pathogen detection technologies in the clinical setting or other surveillance studies.

One benefit of TAC technology is the ability to simultaneously test for numerous pathogens using a relatively small amount of the primary specimen. With 48 wells available for testing, it is necessary to balance the number of targets tested and the number of replicates of each target. Testing of whole blood specimens in this study revealed that numerous positive results likely would have been missed if tested using only two replicates of each assay. It is possible that improved detection for each pathogen is simply due to increasing the proportion of the specimen which is evaluated by testing additional replicates, providing more opportunities for the pathogen-specific nucleic acid to end up in the 1 µL reaction well containing the appropriate oligonucleotides for amplification. Indeed, performing a higher number of assays specific for a given pathogen was previously found to improve detection in blood collected from murine infection models [10]. While increased replicate testing improves pathogen detection in whole blood specimens where pathogen load is commonly low, this approach necessarily reduces the number of targets for which a single specimen can be interrogated. Careful consideration was given to identify the appropriate assays to remove from the blood TAC in order to improve testing for the most critical pathogens of interest in neonates on the modified versions of the ANISA study-specific TACs (Figure 1). Specifically, we increased the number of replicate wells of pathogen targets for which positive specimens were identified and selectively removed assays that failed to identify any positive specimens during pilot phase testing in order to achieve higher confidence in results from a more focused array of pathogen-specific assays. Careful consideration of the unique features and logistical challenges associated with implementing TAC in any setting, whether for clinical use or surveillance, should occur before customizing TACs.

The results reported here indicate that optimization is required when utilizing TAC for pathogen detection in whole blood; our findings may also be applicable to other multiple-pathogen approaches simultaneously evaluating bacterial and viral agents and involving multiple specimen types. Overall, substantial cumulative improvements in Ct values can be realized through optimization of experimental procedures (Figure 5). For example, an initial lysis step prior to the automated nucleic acid extraction procedure improved detection of gram-positive bacterial targets in whole blood as measured by an average decrease in Ct values of six cycles (Figure 2), presumably by facilitating destruction of the peptidoglycan layer and release of cellular contents including nucleic acid. Treatment with lytic enzymes has previously been shown to improve bacterial DNA recovery from blood of neonates [18] and has also been recommended for optimal extraction of nucleic acid from other specimen types [19]–[21]. Based on performance of the selected extraction methods for pathogen detection in respiratory swab specimens, we chose to perform the initial lysis treatment on whole blood specimens only for this study. We selected specimen input and elution volumes in order to achieve a four-fold concentration of nucleic acid from the original specimen, which may result in a theoretical improvement of approximately 1.5 cycles compared to extraction using equal input and elution volumes (Figure 5). Optimization of nucleic acid extraction procedures should be performed for all specimen types since each may provide unique challenges for pathogen detection using TAC or other multiple pathogen testing strategies. Extraction via magnetic bead-based methods, such as the platform used in the current study, has been shown to lessen effects of PCR inhibitors compared to other extraction methods [22]. This may be especially important when testing whole blood specimens with PCR assays. Development and implementation of methods for specific enrichment of pathogen nucleic acid may further improve detection in whole blood specimens. Extraction platform (manual or automated) should also be carefully selected based on site or study-specific needs; reliability, availability of technical support, and extent of training required should be considered when selecting an extraction method for use in a clinical or public health laboratory or in population-based surveillance studies, particularly in resource-limited settings.

Use of TAC or other platforms with individual reaction wells allow the user to customize oligonucleotides for unique assay variations such as asymmetric primer concentrations, locked nucleic acid oligonucleotides, inclusion of more than three oligonucleotides, or other modifications which may be necessary for optimizing performance of individual assays. This is a particular advantage for assays intended to detect multiple species, serotypes, or variants. Although we did not identify any positive effect of increasing overall oligonucleotide concentration on performance of several assays on the TAC format, these data represent only four assays at two different oligonucleotide concentrations each; therefore, limited conclusions can be drawn from this evaluation. Carrying out a full assessment of each assay’s performance at multiple oligonucleotide concentrations was prohibited by cost and logistical challenges, but this comprehensive analysis may reveal target-specific effects. Optimization of oligonucleotide concentration in individual real-time PCR reactions prior to implementation on TAC is recommended. Commercial availability of TACs would obviate the need for this evaluation when applying this technology for clinical or surveillance purposes.

Through direct comparison of Ct values for positive targets in primary specimens, we determined that improved detection can be achieved by selection of an appropriate enzyme formulation. Newer generation enzyme mixes are more robust and may demonstrate improved sensitivity compared to historical enzyme systems; our results show this difference in Ct values may be as large as 10 cycles (Figure 5). This improvement is particularly important in blood specimens where the limit of detection is often significantly higher compared to detection of the same target in other specimen types, in some cases differing by several orders of magnitude. This phenomenon may be due, in part, to inhibitory molecules that can impede pathogen detection. In this case, any incremental increase in sensitivity is valuable. Additionally, enzyme formulations available as a 2× mix, including both systems evaluated in this study, allow for addition of nucleic acid up to approximately one-half of the total reaction volume, which may result in a small improvement in Ct value (approximately one cycle). More highly concentrated enzyme mixes may allow addition of an even greater proportion of nucleic acid to the reaction, perhaps further improving pathogen detection. Additionally, newer enzyme formulations may also exhibit improved stability and simplified use. For example, the Quanta ToughMix enzyme system is a true single-tube reaction mix containing all reagents, including the polymerase and reverse transcriptase enzymes, whereas most historical RT-PCR mixes, including AgPath, require separate storage and addition of enzymes to the mastermix. The stability of the Quanta ToughMix at refrigeration temperatures is an additional benefit, particularly in laboratories where reagent storage space in deep freezers may be limited or unreliable. Minimizing residual *E. coli* DNA and achieving consistency in its concentration in production lots may be another benefit of newer generation enzyme formulations.

While the ability to customize the TAC format is certainly an attractive feature, this brings with it the complex challenge of determining the optimal configuration of assays for each application and producing a customized positive control for all targets in order to ensure confidence in results. In our experience, simply combining DNA or RNA from each organism is not a reliable or feasible option for use as a positive control with the TAC method. To solve this problem, our laboratory previously reported design and performance of a plasmid that can be used to generate RNA transcripts to serve as a positive control for all RT-qPCR reactions on the TAC [17]. A similar solution may be applied to other multiple pathogen detection assays. Furthermore, creating user-friendly databases for storage and analysis of the large number of data points obtained from these multiple-pathogen testing modalities is a challenge requiring collaboration with data management professionals and software programming specialists.

Here we report further optimization for the implementation of TAC technology for pathogen detection in clinical specimens. Despite these technical improvements, other logistical issues impact the widespread implementation of TAC technology for routine use in public health laboratories and population-based surveillance studies. Currently, access is limited due to the lack of a commercially available complete product; therefore, up-front production requirements (preparation of oligonucleotides) and quality control (QC) assessments, which are expensive and labor-intensive, are the responsibility of the consumer. Commercialization of TAC will likely be critical for widespread use in laboratories worldwide. Nonetheless, as the necessary instrumentation and customized TACs become increasingly accessible at public health and clinical laboratories, this technology will inevitably increase the available repertoire of diagnostic tests. To this end, the CDC is actively pursuing initiatives to transfer this technology to state and local public health laboratories for use in outbreak settings. Ultimately, the breadth of testing possible from a small amount of specimen and the relatively rapid time to results provides distinct advantages over existing microbiological and molecular diagnostic methods. Further development and evaluation would be needed in order to meet regulatory standards set by Clinical Laboratory Improvement Amendments (CLIA) or the U.S. Food and Drug Administration (FDA) before this technology may be applied for patient use in the U.S. Still, expansion of multiple-pathogen detection systems such as TAC has the potential to improve patient care on an individual level as well as contribute to the broader challenge of improving public health overall.

In conclusion, our findings demonstrate multiple techniques that can be used to optimize detection by TAC; these approaches may be applicable to other systems that simultaneously detect a range of pathogen types. Such systems are critically needed for outbreaks and clinical situations where rapid results are paramount, especially if a diverse range of pathogens and specimen types need to be considered. Implementation of optimized TAC procedures in the ANISA study will provide our first evaluation of performance of this technology in a large-scale surveillance study. The results will greatly improve our understanding of the pathogens that cause severe illness in neonates in a region of the world where reducing child mortality is a critical need. More broadly, utilization of multiple-pathogen detection approaches such as TAC is likely to increase in the near future and holds promise to improve the overall clinical microbiological evaluation of patient specimens for surveillance, outbreak response, and clinical care.

## Supporting Information

File S1 Contains Table S1, Real-time PCR assays for use on TAC in the ANISA study, and Table S2, Analytical validation of newly developed real-time PCR assays for ANISA study.
(DOCX)
