# Supplementary material for: Optimization of Multiple Pathogen Detection Using the TaqMan Array Card: Application for a Population-Based Study of Neonatal Infection
Source: PLoS One. 2013 Jun 21;8(6):e66183. doi: 10.1371/journal.pone.0066183 (PMC3689704; doi:10.1371/journal.pone.0066183)
Supplement: File S1 — Contains Table S1, Real-time PCR assays for use on TAC in the ANISA study, and Table S2, Analytical validation of newly developed real-time PCR assays for ANISA study. (DOCX) [file pone.0066183.s001.docx]

**Table S1.** Real-time PCR assays for use on TAC in the ANISA study.

| **Organism** | **Target** | **Primer/Probe sequence (5’🡪3’)^a^** | **Conc. (nM)** |
| --- | --- | --- | --- |
| *Toxoplasma gondii* | ssrRNA | F, GGTGGTCCTCAGGTGAT | 1000 |
|  |  | R, CCACGGTAGTCCAATACAGTA | 1000 |
|  |  | P, FAM-ATCGCGTTGACTTCGGTCTGCGAC-BHQ1 | 200 |
| *Staphylococcus aureus* | Glutamate synthase | F, CGGGTTAGGTGAATTGATTGTTTTAT | 1000 |
|  |  | R, CGCATTTGAGCTGAAGTTG | 1000 |
|  |  | P, FAM-TTCCATATGACCACCACGAGTCTTAGCACC-BHQ1 | 200 |
| *Klebsiella pneumoniae* | Diguanylate cyclase | F, TGCAGATAATTCACGCCCAG | 1000 |
|  |  | R, ACCCGCTGGACGCCAT | 1000 |
|  |  | P, FAM-CCACCACGCTCATCTGTTTCGCC-BHQ1 | 200 |
| *Escherichia coli*/*Shigella* spp.^b^ | *uidA* | F, GAGCATCAGGGTGGCTATACG | 500 |
|  |  | R, ATAGTCTGCCAGTTCAGTTC | 500 |
|  |  | P, FAM-TACGGCGTGACATCGGCTTCAAATG-BHQ1 | 100 |
| *Pseudomonas aeruginosa* | *gyrB* | F, GTCTCGGTGGTGAACG | 500 |
|  |  | R, TGGATGTTGCTGAAGGTCTC | 500 |
|  |  | P, FAM-TCCGTCGCCACAACAAGGTCTGGGAA-BHQ1 | 100 |
| *Ureaplasma* spp.^c^ | *ureA* | F, GGTTTAGATACTCACGTTCACTGA | 500 |
|  |  | R, GCTTTTGTACCATCATTCATACCTGT | 500 |
|  |  | P, FAM-CCACCAGCAA"T"AACAGTTGTAATACCACCATC-C6 | 100 |
| *Chlamydia trachomatis* | *ssrA* | F, GGTGTAAAGGTTTCGACTTAGAA | 1000 |
|  |  | R, CGAACACCGGGTCACC | 1000 |
|  |  | P, FAM-ATGCGGAGGGCGTTGGCTGG-BHQ1 | 200 |
| *Acinetobacter baumannii* | *bla*_OXA-51_ | F, TATTTTTATTTCAGCCTGCTCACCTT | 1000 |
|  |  | R, AAATACTTCTGTGGTGGTTGCCTTA | 1000 |
|  |  | P, FAM-TGACTGCTAATCCAAATCACAGCGCTTCA-BHQ1 | 200 |
| *Streptococcus agalactiae* (GBS^d^) | *cfb* | F, GGGAACAGATTATGAAAAACCG | 1000 |
|  |  | R, AAGGCTTCTACACGACTACCAA | 1000 |
|  |  | P, FAM-AGACTTCATTGCGTGCCAACCCTGAGAC-BHQ1 | 200 |
| *Salmonella* spp.^e^ | *ttrRSBCA* | F, CTCACCAGGAGATTACAACATGG | 500 |
|  |  | R, AGCTCAGACCAAAAGTGACCATC | 500 |
|  |  | P, FAM-CACCGACGGCGAGACCGACTTT-BHQ1 | 100 |
| *Neisseria meningitidis* | *sodC* | F, CTGTGAGCCAAAAGAAAAAGAAG | 1000 |
|  |  | R, GATTTGTTGCTGTGCCATCAT | 1000 |
|  |  | P, FAM-CGCAGGCGGTCACTGGGATC-BHQ1 | 200 |

^a^FAM, 6-carboxyfluorescein; BHQ1, Black Hole Quencher 1; C6, 6-Carbon spacer. Each probe was labeled with FAM at the 5’ end and BHQ1 at the 3’ end or at internal base indicated by quotation marks.

^b^Assay detects *E. coli* and *Shigella* spp. (except *S. dysenteriae* serotype 1). Results are only interpretable when Ct ≤ 30.

^c^Assay detects all serogroups of *U. urealyticum* and *U. parvum*.

^d^GBS, Group B *Streptococcus*.

^e^Assay reported previously (12).

**Table S2.** Analytical validation of newly developed real-time PCR assays for ANISA study.

| **Assay** | **Limit of Detection** | **Isolates tested (n)** | **Species, serotypes/serovars, groups tested** |
| --- | --- | --- | --- |
| *T. gondii* | < 1fg | 1 |  |
| *S. aureus* | 100fg-10fg/µL | 11 | MRSA clonal groups USA 100, 200, 300, 400, 500, 800, 1000, Brazilian, EMRSA 15, and ST80 |
| *K. pneumoniae* | 100fg/µL | 2 |  |
| *E. coli* and *Shigella* spp. | 10fg-1fg/µL | 94 | 4 species of *Shigella* |
| *P. aeruginosa* | 100fg-10fg/µL | 7 |  |
| *Ureaplasma* spp. | 1pg/µL | 14 | Serotypes 1-14 of *U. urealyticum* and *U. parvum* |
| *C. trachomatis* | 15 copies/µL | 4 | Serovars D, E, H, and F |
| *A. baumannii* | 10fg-1fg/µL | 1 |  |
| *S. agalactiae* (GBS) | 300fg/µL | 30 | Serotypes 1A, 1B, 2-7, and non-typeable isolates (*n* = 2) |
| *Salmonella* spp. | 1pg/µL | 116 | Serotypes/subspecies I, II, IIIa, IIIb, IV, and V |
| *N. meningitidis* | 1 fg/µL | 9 | Serogroups A, B, C, 29E, W135, X, Y, Z and non-groupable isolates |
